# ASO Author Reflections: Impact of Ipsilateral Supraclavicular Lymph Node Dissection on Breast Cancer

**DOI:** 10.1245/s10434-020-09585-1

**Published:** 2021-02-02

**Authors:** Minhao Lv, Suxia Luo

**Affiliations:** 1 Department of Breast Disease, The Affiliated Cancer Hospital of Zhengzhou University, Zhengzhou, China; 2 Department of Medical Oncology, The Affiliated Cancer Hospital of Zhengzhou University, Zhengzhou, China

## PAST

Breast cancer with ipsilateral supraclavicular lymph node metastasis (ISLNM) has been reclassified from M1 distant disease to N3c locoregional disease due to similar prognosis.^1^ As neoadjuvant chemotherapy (NAC) has become part of the standard of care for patients with locally advanced breast cancer (LABC) and has been proved to be capable of eradicating nodal disease in considerable amounts of patients with axillary lymph node metastases,^2^ the role of surgery nodal dissection in nodal eradication should be re-evaluated. However, little evidence is available regarding the standard ipsilateral supraclavicular treatment strategy (surgery and radiotherapy) for patients with ISLNM. Meanwhile, even though some studies have constructed nomograms to predict the probability of achieving breast or axillary nodal pathological complete response (pCR) after NAC, predictive nomograms focusing on ipsilateral supraclavicular lymph nodes (ISLNs) are still lacking.

## PRESENT

In the current study,^3^ medical records of breast cancer patients at the Affiliated Cancer Hospital of Zhengzhou University, Jiyuan People’s Hospital, and Huaxian People’s Hospital between 21 December 2012 and 15 April 2020 were systematically reviewed. A total of 353 patients with ISLNM were identified. Patients who underwent ipsilateral supraclavicular lymph node dissection (ISLND) had a higher rate of ipsilateral supraclavicular relapse-free survival (ISRFS) but not disease-free survival (DFS) or overall survival (OS). For the low-invasive molecular subtypes, including Luminal A and Luminal B subtypes, the use of ISLND was associated with a higher rate of ISRFS and DFS. For the aggressive molecular subtypes, including Luminal-HER2, HER2+ and Triple-negative subtypes, the use of ISLND was associated with a higher rate of ISRFS.

A nomogram was constructed to calculate the probability of achieving ipsilateral supraclavicular pCR using the following five factors: number of axillary lymph node metastases, breast pCR, size of the ISLN after NAC, number of NAC cycles, and ki67 level.

## FUTURE

This study is based on retrospective data collection, and sample size is relatively small, leading to a non-random patient distribution and only 46 patients receiving radiotherapy alone. This fact might somehow compromise the representativeness of this cohort. Meanwhile, even though efforts have been made to verify our nomogram by reaching out to external data by investigators, verification at the current stage is somehow unachievable given the paucity of related studies and the lack of an extra database. Prospective studies with larger sample sizes are needed in further studies in order to better investigate the impact of ISLND on breast cancer patients and to verify or improve this nomogram.
